# The 10–23 DNAzyme in Biosensing and Diagnostics: Applications, Challenges, and Future Directions

**DOI:** 10.1002/anie.7876181

**Published:** 2026-02-28

**Authors:** Connor Nurmi, Jake Brill, Sanne Roumans, Amal Mathai, John D. Brennan, Yingfu Li

**Affiliations:** ^1^ Department of Biochemistry and Biomedical Sciences McMaster University Hamilton Ontario Canada; ^2^ Biointerfaces Institute McMaster University Hamilton Ontario Canada; ^3^ Schulich School of Medicine & Dentistry Western University London Ontario Canada

## Abstract

Catalytic DNA molecules (DNAzymes) have garnered increasing attention as components of biosensing and diagnostic platforms due to their simplicity, programmability, and cost‐effectiveness. Among them, the 10–23 DNAzyme remains the most widely used RNA‐cleaving DNAzyme, combining high catalytic efficiency with broad adaptability across diverse sensor architectures. Despite these advantages, its performance can be significantly hindered by suboptimal reaction temperature, low Mg^2+^ concentrations, nuclease‐rich biological matrices, and restricted accessibility to structured RNA targets. Such limitations have impeded its widespread adoption in simple, robust point‐of‐care formats. This review examines the integration of the 10–23 DNAzyme into contemporary biosensing and diagnostic systems—including colorimetric, fluorescent, electrochemical, electrochemiluminescent, and intracellular sensors—highlighting both direct and regulated activation strategies and the dual role of 10–23 as a molecular recognition element and signal reporter. We also discuss key challenges in catalytic performance, stability, assay workflow, and clinical validation, as well as emerging solutions such as chemical modifications, nanoparticle‐based protection, and advanced sensor architectures. Together, these insights outline the current landscape and future opportunities for advancing the 10–23 DNAzyme toward next generation biosensing and diagnostic applications.

## Graphical abstract

This review focuses on the 10–23 DNAzyme in diagnostics, spanning unregulated and regulated target‐recognition modes, and their functionalization across colorimetric, fluorescent, electrochemical, electrochemiluminescent, and intracellular biosensing strategies. Key real‐world barriers to simple point‐of‐care use are highlighted, along with emerging solutions that improve performance, stability, and workflow robustness to accelerate practical translation.

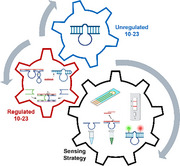

## 1 Introduction

### 1.1 The Role of Enzymes in Diagnostics and Biosensing

Enzymes are indispensable tools in diagnostics and biosensing, where their high specificity and catalytic efficiency enable rapid and accurate detection of biomolecules. For example, glucose oxidase is central to personal glucose meters for diabetes management [1, 2], where the enzyme catalyzes the oxidation of glucose to produce a measurable, concentration‐dependent electrochemical current [3, 4]. Enzymes such as horseradish peroxidase and alkaline phosphatase catalyze colorimetric reactions fundamental to enzyme‐linked immunosorbent assays (ELISA) [5, 6]. These examples underscore the pivotal role of enzymes in creating diagnostic tools that are both sensitive and user‐friendly.

Despite these advantages, protein‐based enzymes can be limited by low stability and high cost, with a relatively narrow substrate range, restricting their use in point‐of‐care (POC) diagnostics. In response, researchers have pursued alternative catalytic systems, including the directed evolution of novel protein enzymes [7] and, more recently, nucleic‐acid‐based catalysts such as ribozymes [8, 9] and DNAzymes [10, 11, 12, 13]. The latter offers several advantages over traditional enzymes, including simpler synthesis, lower cost, high stability, and exceptional programmability.

### 1.2 Discovery and Characterization of the 10–23 DNAzyme

DNA, long recognized as a storehouse of genetic information, can also be engineered to perform catalytic functions through in vitro selection [8, 14, 15]. This process identifies specific catalytic sequences from a random DNA pool via iterative rounds of selection and amplification. Over the years, in vitro selection has yielded DNAzymes capable of diverse catalytic activities, ranging from cleavage of RNA [10, 13] and DNA [16] to DNA ligation [12], porphyrin metalation [17], and amino acid phosphorylation [18]. The DNAzyme database (DNAmoreDB) provides a comprehensive catalog of such catalytic molecules [19].

The first DNAzyme, GR‐5, was discovered in 1994 via in vitro selection and cleaved RNA in a Pb^2+^‐dependent manner [10]. Since then, numerous RNA‐cleaving DNAzymes (RCDs) have been identified, activated by diverse targets such as metal ions, bacteria, mammalian cells, and proteins [20, 21]. Among them, the 8–17 and 10–23 DNAzymes, reported by Santoro and Joyce in 1997 [13, 22], remain the most efficient and widely studied.

**FIGURE 1:**
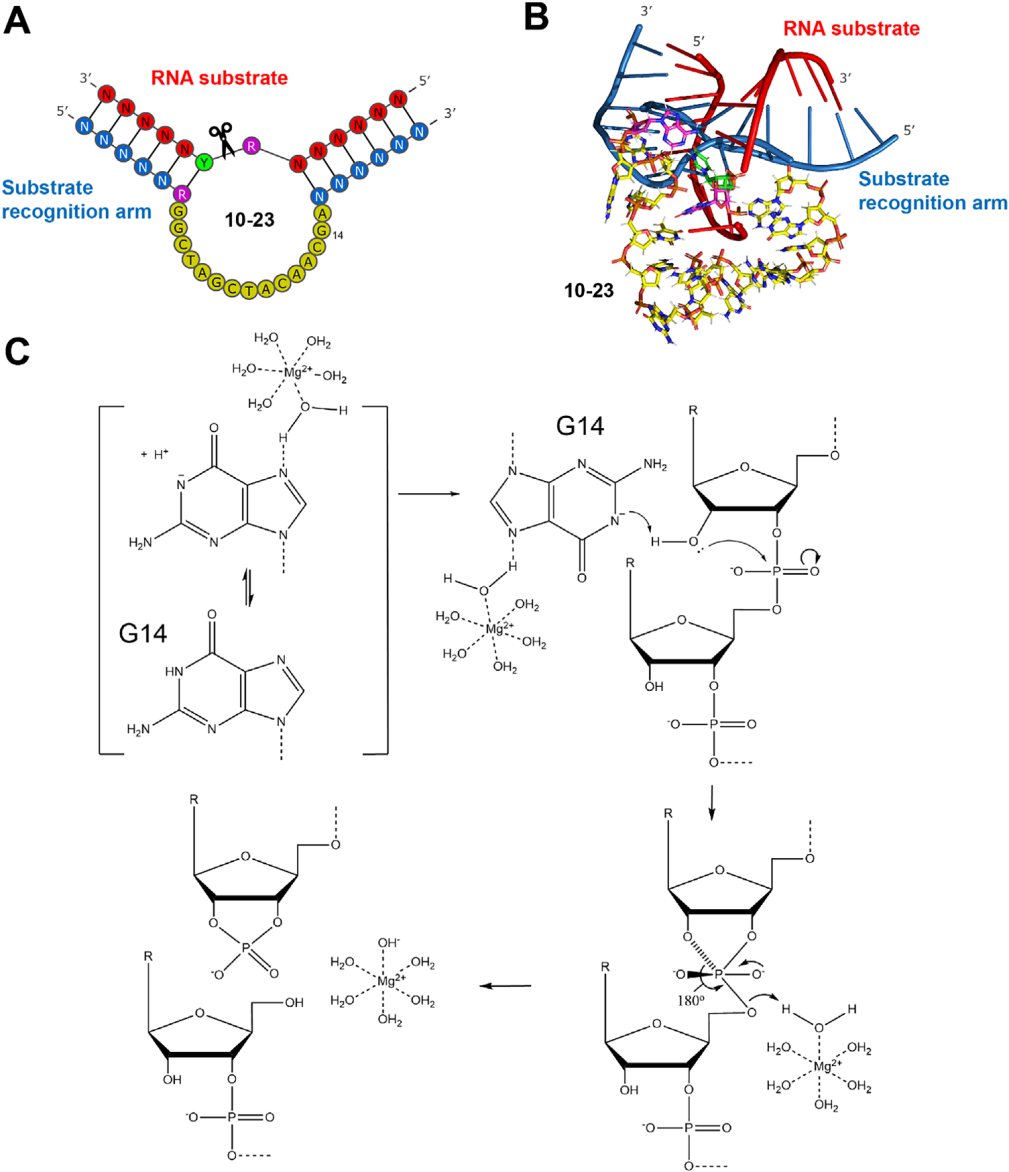
Structural and mechanistic features of the 10–23 DNAzyme. (A) Secondary structure of the 10–23 DNAzyme. The RNA substrate is shown in red with the binding arms in blue. The catalytic core of 10–23 is in yellow, with purines (R) and pyrimidines (Y) colored pink and green, respectively. (B) Tertiary structure of the 10–23 DNAzyme with the same color scheme as in (A), made using PyMOL [37]. (C) Likely catalytic mechanism of the 10–23 DNAzyme based on recent NMR structural data and MD simulations, suggesting that hydrated Mg^2^^+^ coordination with N7 of G14 within 10–23 promotes proton abstraction and in‑line attack, illustrating RNA cleavage with a pseudo‐first order catalytic rate constant (*k*_obs_) up to 12 min^−1^ under fully optimized conditions.

Structurally, the 10–23 DNAzyme is comprised of a 15‐nucleotide catalytic core sequence (5′‐GGCTAGCTACAACGA‐3′) that cleaves RNA in both *cis* and *trans* (Figure 1A,B). In the *cis* configuration, the catalytic and substrate domains are part of the same continuous strand, whereas in the *trans* configuration, they exist as separate oligonucleotides. Catalysis proceeds in a Mg^2^^+^‐dependent manner—though other divalent cations (e.g., Ca^2+^, Mn^2+^) can also promote activity. Lacking canonical base‐pairing within its catalytic core, 10–23 forms a dynamic interaction network that enhances Mg^2+^ binding and promotes efficient folding [23, 24]. The substrate recognition arms located on both sides of the catalytic core can be re‐engineered to target a wide range of RNA sequences. The enzyme exhibits the highest activity at AU and GU junctions, where cleavage occurs between unpaired purines and paired pyrimidines [25].

Initial kinetic data obtained by Breaker and colleagues suggested that the 10–23 DNAzyme used Mg^2^^+^ to coordinate the substrate 2′‑hydroxyl for nucleophilic attack under physiological conditions, forming a di‑anionic pentacoordinate intermediate and yielding 2′,3′‑cyclic phosphate and 5′‑hydroxyl products [26]. However, more recent NMR‑guided molecular dynamics (MD) simulations show that G14 of the 10–23 DNAzyme contacts the substrate's 2′‑oxygen 5‐fold more frequently compared to Mg^2^^+^, suggesting hydrated Mg^2^^+^ coordination with N7 of G14 [27] promotes proton abstraction and in‑line attack [28, 29]. These findings support a more likely, revised catalytic model for the 10‑23 DNAzyme, shown in Figure 1C.

Although the 10–23 DNAzyme has been widely explored for therapeutic applications—supported by numerous clinical trials [30, 31, 32] and reviews [33, 34, 35, 36]—its potential in biosensing has also attracted increasing attention. Acting as both a molecular recognition element (MRE) and a signal reporting element (SRE), the 10–23 DNAzyme represents a versatile platform for developing cost‐effective, sensitive, and programmable diagnostic tools.

### 1.3 Overview of Biosensors Involving 10–23

Biosensors are analytical devices that integrate a biological recognition element with a physicochemical transducer to detect specific analytes. Broadly used in both environmental testing and clinical diagnostics, biosensors can rapidly and accurately determine the presence of target molecules in a sample. A typical biosensor comprises four main components: (1) the sample input, where the analyte is introduced and, in some cases, partially processed; (2) an MRE, which binds the target; (3) an SRE, which converts binding events into a detectable signal; and (4) a final readout displaying the results.

Classic biosensors—such as glucose meters and home‐pregnancy tests—employ enzymes or antibodies as the MRE, respectively, ensuring high specificity and sensitivity. However, modern biosensing platforms have integrated alternative biorecognition elements such as DNA aptamers and DNAzymes as the MRE, combining them with innovative SRE and transduction mechanisms. This integration has substantially expanded the scope of biosensor design while maintaining precise detection of biological analytes.

Within this evolving framework, the 10–23 DNAzyme has emerged as a powerful component, capable of functioning as both an MRE and an SRE due to its robust catalytic activity, inherent programmability, and adaptability across multiple assay formats. Consequently, 10–23 DNAzyme‐based sensors can detect a wide array of targets—from genomic RNA to proteins and small molecules—using diverse detection modalities such as colorimetry, fluorimetry, electrochemistry, lateral‐flow assays, and microfluidics.

**FIGURE 2:**
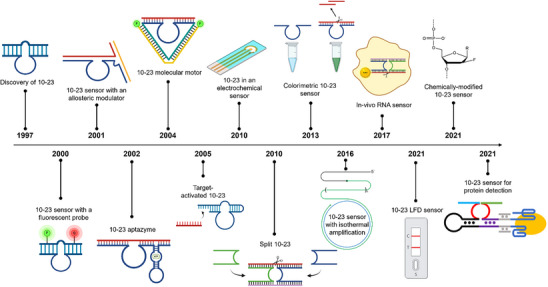
Timeline of key advances in 10–23 DNAzyme‐based biosensors. Major milestones in the development of 10–23 DNAzyme sensors are shown, beginning with its discovery by Santoro and Joyce in 1997 [13], and encompassing representative assay formats, structural and chemical modifications, and performance improvements achieved over time. These innovations demonstrate the expanding versatility of the 10–23 DNAzyme for diverse diagnostic applications.

Figure 2 outlines the key milestones in the evolution of 10–23 DNAzyme‐based sensors and detection assays since its discovery, highlighting the diverse assay formats and sequence modifications that have broadened its applicability. Despite significant progress, no comprehensive review has yet synthesized these developments. This article therefore aims to examine the role of the 10–23 DNAzyme in diagnostic assays, emphasizing sensing strategies, key performance challenges, and emerging solutions to advance its integration into next generation biosensing platforms.

## 2 Direct Target Recognition by Unregulated 10–23 DNAzymes

In this review, we define unregulated 10–23 DNAzymes as those operating in their original, unmodified form, where catalysis proceeds spontaneously upon substrate binding without the involvement of external triggers or conformational switches. These biosensors therefore directly couple target recognition and catalytic cleavage, using the enzyme both as the MRE and the catalyst. Because the substrate‐recognition arms of 10–23 can be readily reprogrammed, unregulated 10–23 DNAzyme sensors are particularly well suited for detecting a wide range of RNA targets and have been widely adopted for nucleic‐acid‐based diagnostics.

By contrast, regulated 10–23 DNAzymes are engineered to remain inactive until specific triggers—such as aptamer binding, split‐core assembly, chemical release, or toehold‐mediated strand displacement—initiate catalytic cleavage. These systems enable conditional activation, dual or cooperative recognition, and enhanced specificity, allowing detection of a broader range of analytes compared to their unregulated counterparts. Further details of regulated 10–23 DNAzyme architectures are discussed in Section 3.

### 2.1 Unregulated 10–23 DNAzyme Sensors

Under optimal reaction conditions (37°C, 20 mM Mn^2+^, 50 mM HEPPS pH 7.4, 150 mM NaCl, and a short RNA target), the 10–23 DNAzyme demonstrated a single‐turnover catalytic rate constant (*k*_obs_) exceeding 12 min^−1^ [22]. Later studies confirmed it could cleave longer RNAs such as rat c‐myc mRNA (2200‐nt) and HPV16 E6 mRNA (540‐nt), with rates ranging from 0.02 to 0.21 min^−1^—values that, although slower, remain sufficient for many diagnostic applications [38]. Early diagnostic assays used polyacrylamide gel electrophoresis (PAGE) to confirm the ability of 10–23 to cleave RNA from real samples in a sequence‐specific manner, with targets including vascular endothelial growth factor receptor (VEGFR2) mRNA [39] and 16S rRNA from *Sphaerotilus natans* [40] in wastewater samples. However, PAGE‐based assays are not suitable for POC detection.

**FIGURE 3:**
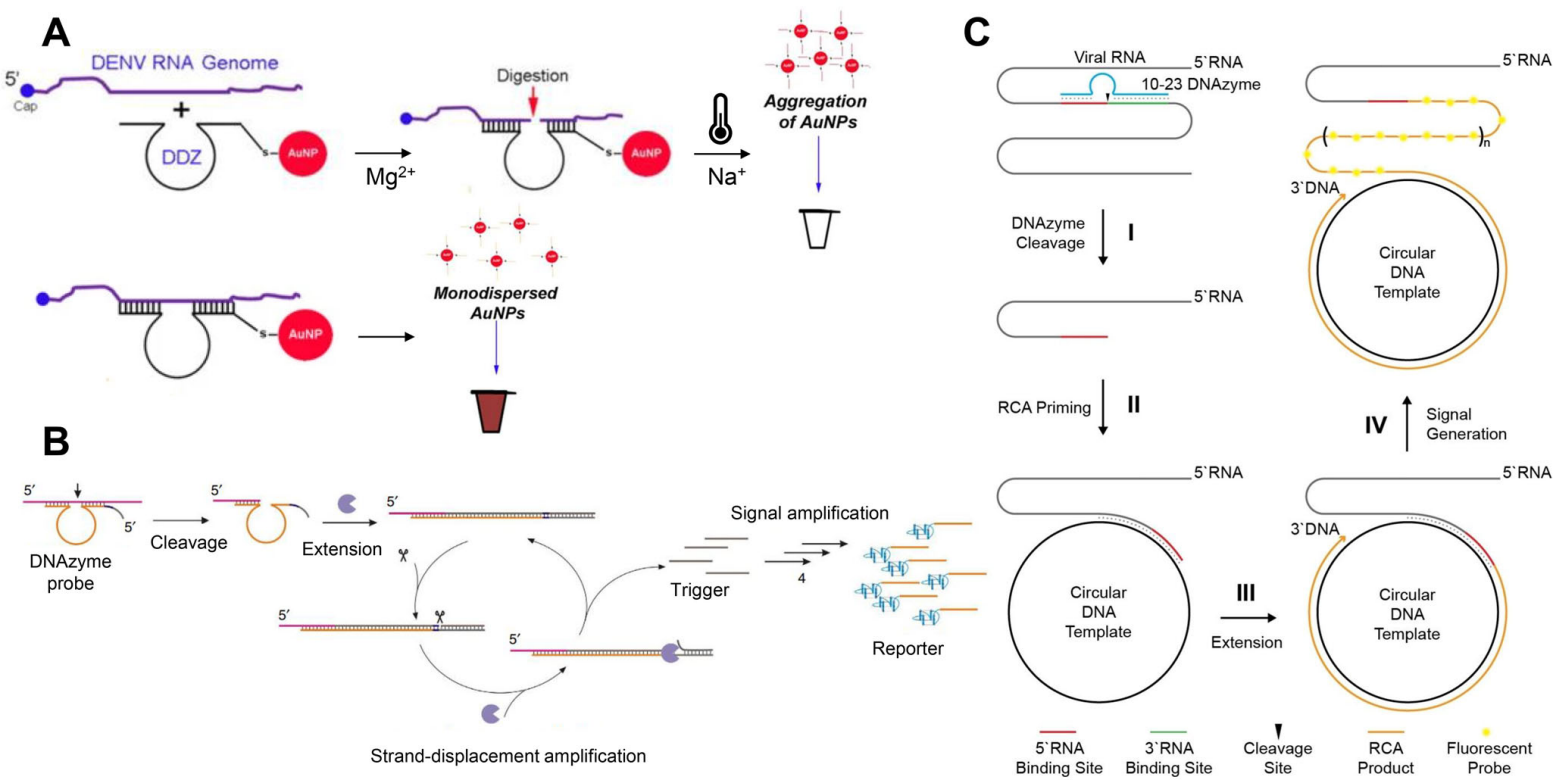
Sensors that use unregulated 10–23 DNAzymes. (A) Solution‐based colorimetric sensor for dengue virus (DENV) RNA detection, where unregulated 10–23 DNAzymes (DDZ) functionalized to AuNPs disperse upon cleavage of DENV RNA, turning the solution clear. Adapted from Ref. [41] with permission. (B) Solution‐based unregulated 10–23 DNAzyme sensor triggers strand displacement amplification (SDA) upon target RNA binding, forming G‐quadruplex DNAzymes that yield a color change in the presence of hemin, peroxide, and ABTS. Reproduced from Ref. [42] with permission. (C) Fluorogenic sensor coupling unregulated 10–23 DNAzyme cleavage of SARS‐CoV‐2 RNA with rolling circle amplification (RCA). Reproduced from Refs. [43, 44] with permission.

Recent innovations have introduced colorimetric detection strategies that better align with practical diagnostic applications. An early representative example is the gold nanoparticle (AuNP)‐based colorimetric assay employing an unregulated 10–23 DNAzyme, which detects dengue virus (DENV) mRNA through DNAzyme‐functionalized AuNP aggregation, producing a visible red‐to‐clear color change under mild conditions (Figure 3A) [41]. The assay used diluted DENV samples from clinical saliva, achieving a limit of detection (LoD) of 60 pM (Table 1) with a total assay time of 30 min and incubation at 37°C. However, several incubation and pipetting steps were required, limiting its applicability for POC testing.

**TABLE 1:** Summary of unregulated 10–23 DNAzyme biosensors.

10‐23 DNAzyme Type	Assay type	Target	Amplification step?	Clinical samples used?	Assay time	Assay temperature	LoD	Ref.
Unregulated	Optical	DENV RNA	No	No	30 min	37°C	60 pM	[41]
Unregulated	Fluorescence	Let‐7a miRNA	SDA	No	3.5 h	37°C	69 aM	[42]
Unregulated	Fluorescence	SARS‐CoV‐2 mRNA	Quasi‐exponential RCA	Patient Saliva	3 h	23°C	500 aM	[43, 44]
Unregulated	Fluorescence	LSDV DNA	RPA	Cattle	3 h	37°C	∼ 1 aM	[45]

Another unregulated 10–23 DNAzyme design incorporated a strand‐displacement amplification (SDA) reaction step to improve assay sensitivity (Figure 3B) [42]. In this system, cleavage of synthetic target miRNA or mRNA by 10–23 triggered SDA, during which the cleavage products were extended by DNA polymerase and nicked by a nicking endonuclease, releasing additional trigger molecules and forming G‐quadruplex structures that produced a green color in the presence of hemin, peroxide, and ABTS. While SDA significantly enhanced assay sensitivity, achieving an LoD of 69 aM, it required a 3.5 h incubation at 37°C—much longer than the 30 min AuNP‐based colorimetric assay for DENV. Moreover, reagents such as peroxide and ABTS have limited shelf lives, further constraining their utility in commercial biosensor applications.

### 2.2 Improving Target Recognition by 10–23

#### 2.2.1 Targeting Large Structured Genomic RNAs

In the previous examples, unregulated 10–23 DNAzymes were designed to directly recognize and cleave an RNA target. However, complex secondary and tertiary structures within larger RNA molecules (mRNA, genomic RNA) often hinder the accessibility of the target site to 10–23, posing a significant challenge for direct target recognition and thereby limiting the development of these sensors. A sensing strategy developed by our group highlights this problem, using large, structured RNA (lsRNA) genes from SARS‐CoV‐2 as model targets.

In our design, 10–23 DNAzymes targeted SARS‐CoV‐2 lsRNA, generating shorter, cleaved RNA fragments that functioned as primers for rolling circle amplification (RCA), which produced concatenated DNA products detected fluorometrically (Figure 3C) [43]. To optimize the sensor, 230 DNAzymes were screened across the SARS‐CoV‐2 genome, with 34 exhibiting high cleavage efficiency, defined as over 20% cleavage of the lsRNA transcript after 10 min. Among these, only 8 produced fragments capable of initiating RCA, with only one system able to successfully identify SARS‐CoV‐2 positive and negative patient saliva samples. Overall, the sensor achieved an LoD of 500 aM within 3 h at room temperature but required a fluorescence reader and multiple sample‐processing steps, making it suitable only for laboratory‐based testing.

This limited success underscored a major challenge: extensive secondary and tertiary structures in genomic RNA hinder the 10–23 DNAzyme substrate recognition arms from accessing their target purine‐pyrimidine cleavage sites. The resulting cleavage fragments also maintained structural elements that obstructed circular DNA template (CDT) binding, affecting primer–CDT annealing and reducing RCA efficiency and overall diagnostic performance.

Several strategies have been developed to improve accessibility to structured RNAs for biosensing applications. One approach involves the use of antisense DNA oligonucleotides (ASOs) to rearrange structural elements [46]. These ASOs bind to upstream and downstream regions, restructuring the RNA to restore or enhance DNAzyme activity. This strategy has been shown to increase the observed cleavage rate (*k*_obs_) of otherwise poorly active 10–23 DNAzymes by up to 2000‐fold. Mechanistic validation using in‐line probing confirmed that ASOs disrupt secondary structures at the DNAzyme target site within lsRNA substrates.

The ASO strategy also enhances the efficiency of downstream amplification reactions initiated by 10–23 cleavage products. In a recent study from our group, ASOs were used to promote hybridization of lsRNA cleavage products to CDTs to initiate RCA (termed ASO‐assisted RCA), employing upstream ASOs to remodel RNA structures and expose the CDT‐binding region [44]. Across five distinct DNAzyme–CDT systems targeting different regions of the SARS‐CoV‐2 genome, ASOs significantly improved CDT binding and amplified RCA signals—by as much as 70‐fold. In clinical saliva samples, ASO‐RCA achieved 100% sensitivity and up to 97.5%–100% accuracy across all systems.

Beyond ASOs, xeno nucleic acids (XNAs) [47, 48, 49] have also been employed to improve accessibility of lsRNA to 10–23 DNAzymes. XNAs with 2′ ribose modifications demonstrated increased RNA binding strength compared to DNA when incorporated into the substrate recognition arms of the 10–23 DNAzyme, leading to higher activity. Examples of these modifications include 2′‐deoxy‐2′‐fluoro‐arabinonucleic acid (FANA) [50, 51], 2′‐*O*‐methyl RNA (2′‐OMe) [52], and locked nucleic acids (LNA) [47].

One study reported that LNA‐modified 10–23 DNAzymes cleaved 68% of a 2904‐nt rRNA target within 1 h, whereas the unmodified enzyme showed no detectable activity [47]. More recently, our group demonstrated that combining different XNA patterns within the substrate recognition arms of 10–23 can further enhance lsRNA accessibility compared to using a single XNA type alone. One XNA‐modified 10–23 DNAzyme construct, XdZ‐2 cleaved lsRNA with an ∼82‐fold improved rate compared to FANA‐modified 10–23 and an ∼135‐fold improvement compared to unmodified 10–23 [53]. More detailed investigations regarding XNAzyme kinetics can be found in several excellent research articles [51, 54, 55].

For biosensing, the increase in lsRNA cleavage activity by XNAzymes can enhance sensing performance compared to unmodified 10–23 DNAzymes. One FANA‐modified 10–23 DNAzyme, X10‐23 [56], was incorporated into a fluorometric sensor for the detection of SARS‐CoV‐2 genomic RNA. Following a preliminary target amplification step, X10‐23 enabled higher sensitivity compared to the DNA version, detecting as low as 500 aM of target, while the original 10–23 produced only a minimal fluorescence signal with a target concentration of 5 fM [57] (more discussion is provided in the subsequent sections). It is important to note, however, that both ASO and XNA strategies involve trade‐offs in cost and synthesis complexity, which must be balanced against their performance gains in biosensor development.

#### 2.2.2 Targeting Genomic DNAs

A major limitation of all RCDs, including 10–23, is their inability to directly target and cleave genomic DNA, which restricts their utility in diagnostic applications. Many clinically important pathogens possess DNA genomes, encompassing all bacteria and roughly 56% of viruses [58, 59], including hepatitis B virus, herpesvirus and adenovirus. To overcome this limitation, an unregulated 10–23 DNAzyme can be coupled with an MRE that recognizes DNA, such as CRISPR‐Cas12a, to enable genomic DNA detection.

In one example, a fluorometric sensor for detecting lumpy skin disease virus (LSDV)—a double‐stranded DNA virus—was developed by coupling CRISPR–Cas12a with 10–23. After extracting LSDV genomic DNA using a commercial kit (QIAamp DNA Mini Kit) and amplifying it via recombinase polymerase amplification (RPA), a complementary CRISPR RNA (crRNA) recruited Cas12a endonuclease to cleave an unregulated 10–23 DNAzyme, thereby inactivating it. In this signal turn‐off design, the deactivated 10–23 DNAzyme was unable to cleave a Baby Spinach RNA aptamer, designed to generate fluorescence in the presence of a fluorophore [45]. The assay detected clinical cattle samples with an LoD of ∼1 aM within 3 h at 37°C, but required extensive sample processing and long incubation times, making it incompatible with POC testing.

In addition to genomic DNA detection, coupling 10–23 target recognition with the high cleavage efficiency of the CRISPR–Cas system has proven to be an effective strategy for developing sensors capable of detecting a wide range of analytes, including small molecules [60] and proteins [61], which are discussed in later sections.

## 3 Sensing With Regulated 10–23 DNAzymes

Regulated 10–23 DNAzymes are designed to remain catalytically inactive until specific molecular or environmental triggers activate cleavage. These triggers, such as aptamer binding, split‐core assembly, chemical release, or toehold‐mediated strand displacement, enable conditional control over catalysis, improving specificity and allowing detection of a broader range of analytes compared with unregulated systems.

Biosensors employing regulated 10–23 DNAzymes often achieve higher sensitivity, enhanced selectivity, or context‐dependent activation, depending on the regulatory mechanism used. However, these advantages may come with greater design complexity and longer assay times, necessitating careful optimization for practical implementation, particularly in settings that must meet the World Health Organization ASSURED criteria (Affordable, Sensitive, Specific, User‐friendly, Rapid and robust, Equipment‐free and Deliverable to end‐users) [62].

Regulated 10–23 DNAzyme biosensors have been developed in several formats, each offering distinct mechanisms for controlling catalytic activity. The following sections describe the major subclasses of regulated 10–23 DNAzymes, beginning with allosterically modulated designs in Section 3.1, followed by oligonucleotide split systems (or MNAzymes) in Section 3.2 and target‐activated configurations in Section 3.3, with their integration into amplification schemes discussed in Section 3.4.

### 3.1 Allosterically Modulated 10–23 DNAzymes

Among the different types of regulated 10–23 DNAzymes, allosterically modulated designs introduce an additional oligonucleotide element to control RNA‐cleavage activity, acting as the target molecule itself or as a recognition domain. For example, an aptamer that undergoes structural rearrangement upon ligand binding to activate catalysis. Such designs enable conditional control over DNAzyme activity and are broadly categorized into two representative mechanisms: “three‐way” modulation and aptazyme regulation, which are described below.

#### 3.1.1 Three‐Way Modulation

**FIGURE 4:**
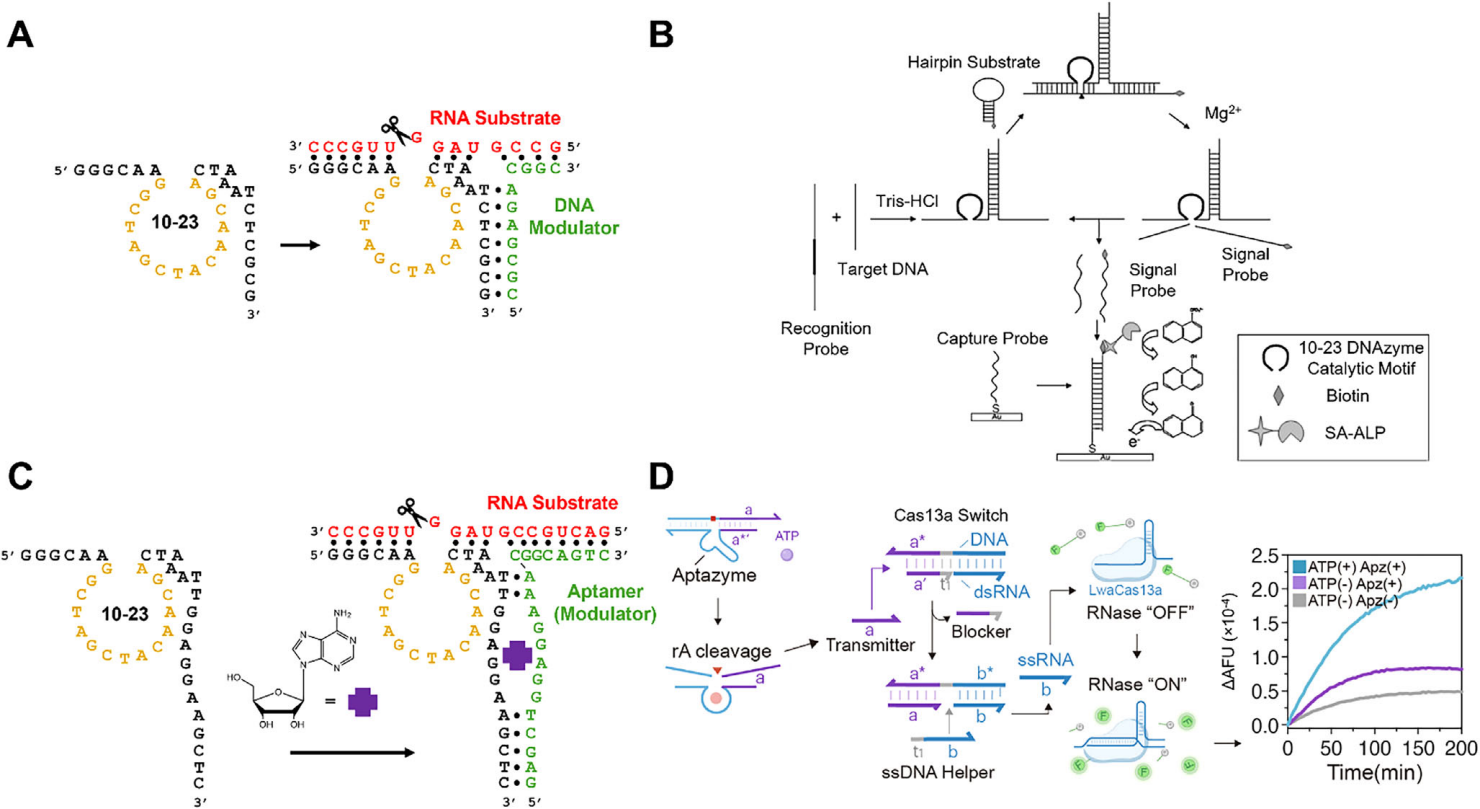
Two types of allosterically modulated 10–23 DNAzymes. (A) Schematic of 10–23 with a DNA oligonucleotide allosteric modulator. (B) Three‐way modulated 10–23 DNAzyme for electrochemical detection of a synthetic DNA target. Reproduced from Ref. [64] with permission. (C) Schematic of 10–23 with an aptamer allosteric modulator. (D) 10–23 with aptamer modulation for fluorescent detection of ATP using CRISPR‐Cas13a activation. Adapted from Ref. [60] with permission.

A three‐way modulation strategy involves the formation of a three‐way junction between the 10–23 DNAzyme, the RNA substrate, and a nucleic acid target acting as the modulator (Figure 4A). In this design, the DNA modulator regulates DNAzyme activity through partial recognition of the RNA substrate [63]. In the absence of the DNA modulator, the RNA substrate cannot hybridize efficiently to the DNAzyme and is not cleaved. When the DNA modulator is present, it promotes stable DNAzyme‐substrate hybridization through formation of a three‐way junction between the DNAzyme and RNA substrate, facilitating subsequent cleavage of the substrate by the DNAzyme.

To date, only one example of a three‐way modulation design using the 10–23 DNAzyme has been reported, employing a biotinylated hairpin RNA substrate for electrochemical detection of a synthetic DNA target. In the presence of the DNA target, a three‐way junction formed between the target, the 10–23 DNAzyme, and the biotinylated hairpin RNA substrate, resulting in substrate cleavage and subsequent hybridization to a capture probe conjugated to a gold electrode (Figure 4B) [64]. The captured biotinylated fragment bound a streptavidin–alkaline phosphatase (ST‐ALP) reporter, enabling dephosphorylation to produce a measurable change in current. The design achieved a limit of detection (LoD) of 50 fM within 4 h at 37°C, without an amplification step (Table 2).

**TABLE 2:** Summary of three‐way modulated 10–23 DNAzyme and 10–23 aptazyme biosensors. *This aptazyme is based on the 10–23 aptazyme but does not contain the 10–23 core sequence.

10–23 DNAzyme Type	Assay type	Target	Amplification step?	Clinical samples used?	Assay time	Assay temperature	LoD	Ref.
Three‐way	Electrochemical	Synthetic DNA	No	No	4 h	37°C	50 fM	[64]
Aptazyme*	Fluorescence	Adenosine	No	No	30 min	23°C	1.4 µM	[67]
Aptazyme	Fluorescence	Adenosine	No	No	2.5 h	37°C	0.16 µM	[60]

Although the three‐way modulation strategy has been successfully demonstrated with other DNAzymes [65, 66], its application to the 10–23 DNAzyme remains limited. This may be because the design is largely restricted to short nucleic acid targets (approximately 25‐nt, as in the above example), since longer and more structured RNAs (e.g., mRNAs or genomic RNAs) are not easily compatible with junction formation. In addition, this format is less suitable for POC diagnostics, as multiple hybridization and washing steps were required.

#### 3.1.2 Aptazymes

Beyond simple DNA modulators, aptamer domains, which selectively bind small molecules or proteins, can be fused to the 10–23 catalytic core to create an “aptazyme”. In this configuration, the DNAzyme remains inactive until the aptamer binds its cognate ligand, inducing a conformational rearrangement that activates the catalytic core in a manner analogous to the three‐way modulation mechanism.

The initial 10–23 aptazyme design incorporated an ATP‐binding aptamer [68] to modulate 10–23 DNAzyme activity for the detection of adenosine (Figure 4C) [69]. The aptamer, which functions as a DNA modulator analogous to the three‐way modulation strategy, is positioned on one of the substrate‐recognition arms, where ATP binding induces a conformational change that realigns the RNA substrate for cleavage by the 10–23 catalytic core. Incorporating this design into a sensor, substrate cleavage by the 10–23 aptazyme was coupled with CRISPR‐Cas13a activation for ATP detection (Figure 4D) [60]. Upon ATP binding, the 10–23 aptazyme cleaved the embedded RNA linkage of a non‐fluorescent DNA substrate, activating a Cas13a “switch” module that displaced a crRNA molecule and facilitating Cas13a‐mediated cleavage of a fluorescent probe. This assay reported an LoD of 0.16 µM, detected ATP in spiked human serum samples within 2.5 h at 37°C, and demonstrated 95% clinical accuracy using five samples, although it required a fluorometer and multiple reaction and sample‐preparation steps.

The use of CRISPR‐Cas13a can be critical for improving detection sensitivity. In an earlier example, an ATP aptazyme sensor was designed based solely on the original 10–23 aptazyme architecture, without CRISPR‐Cas13a activation [67]. In this design, the presence of the adenosine targets induced aptamer folding and subsequent cleavage of the embedded RNA linkage, liberating a fluorophore from its quencher and generating fluorescence only under target‐bound conditions. The sensor detected adenosine in processed human serum within 30 min at room temperature but produced an LoD of 1.4 µM, approximately 10‐fold higher than the Cas13a‐enhanced system. For reference, circulating adenosine typically exists below micromolar levels, rendering this design unsuitable for practical ATP detection [70].

As with the three‐way design, 10–23 aptazymes have not been widely adopted as biosensors. This may be due to the limited availability of aptamers capable of allosterically regulating 10–23 catalysis, or a preference for alternative 10–23 aptazyme configurations—such as antibody conjugated or multiplexed 10–23 DNAzymes—which will be discussed in subsequent sections. Thus far, adenosine detection remains the only diagnostic application of the 10–23 aptazyme. However, as more aptamers become available, new 10–23 aptazyme‐based biosensors are likely to emerge.

Beyond the 10–23 DNAzyme, several aptazyme sensors have been developed using other RCDs for diverse analytes. For example, Pb^2+^‐dependent 8–17 and GR‐5 aptazyme sensors have been reported for detection of IFN‐γ [71] and chlorpyrifos ethyl pesticide [72], respectively. However, the requirement of Pb^2+^, a toxic heavy metal, makes such systems undesirable for commercial applications.

Additional RCD‐based aptazyme sensors have been generated via in vitro selection, in which a library containing a random sequence domain is incubated with the target to evolve a responsive construct. This approach has been widely used for pathogenic bacterial detection, with 16 RCD aptazymes reported to date [73]. To expand the versatility of 10–23 aptazyme sensors to other targets, in vitro selection libraries can be designed with the 10–23 catalytic core linked to a random‐sequence target‐binding domain, providing a path toward discovering new allosterically regulated 10–23 aptazymes.

### 3.2 Multicomponent 10–23 DNAzymes

**FIGURE 5:**
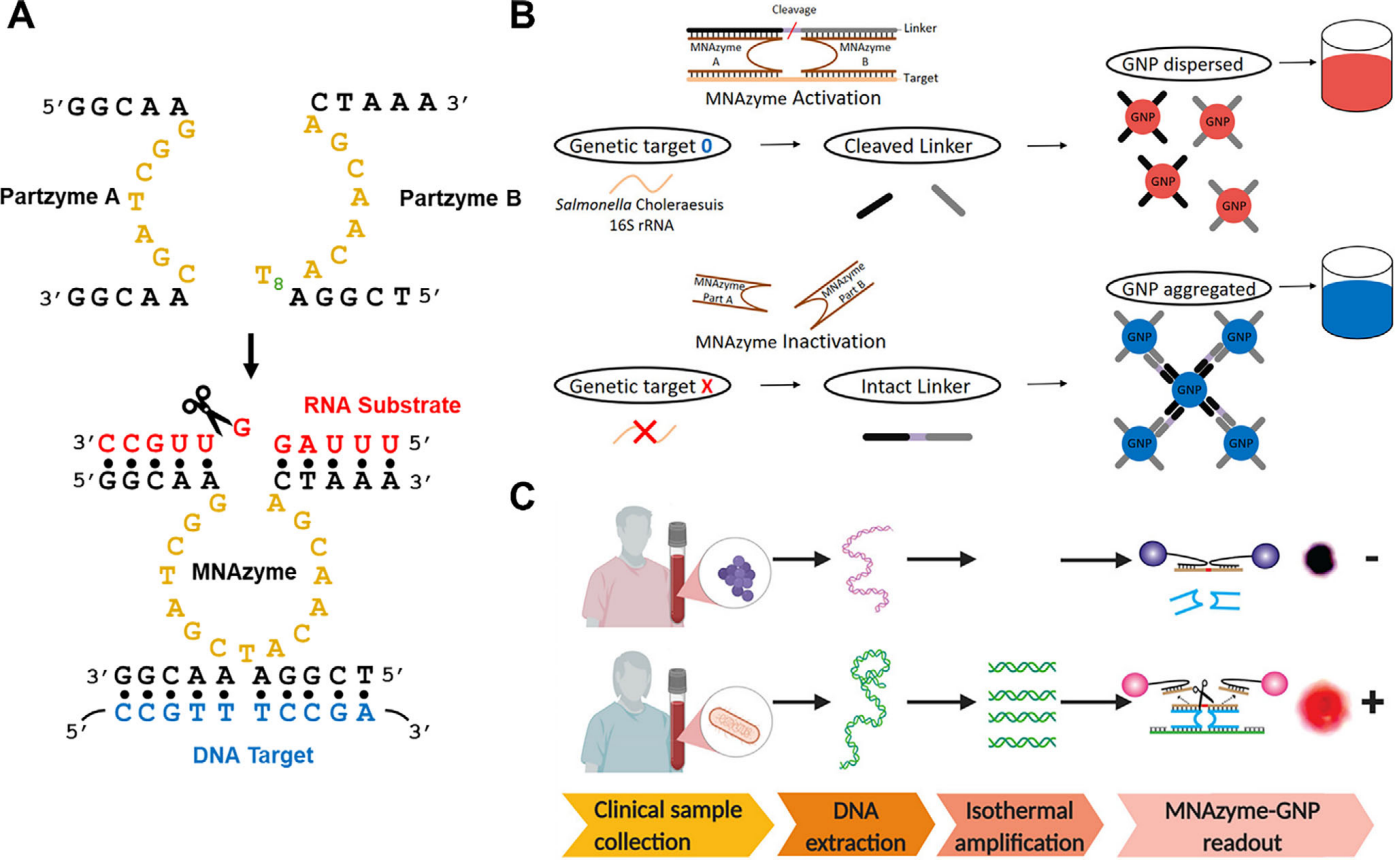
Schematic of the multicomponent DNAzyme (MNAzyme), which forms an active 10–23 catalytic core in the presence of an oligonucleotide target. (A) Assembly of partzyme A and partzyme B into a functional MNAzyme through target‐induced hybridization and subsequent cleavage of an RNA substrate. (B) Colorimetric MNAzyme sensor using AuNPs for *Salmonella choleraesuis* 16S rRNA detection. Reproduced from Ref. [82] with permission. (C) Similar AuNP‐based colorimetric MNAzyme sensor for methicillin‐resistant *Staphylococcus aureus* (MRSA) detection in patient samples. Reproduced with permission from Ref. [85]. Copyright 2025, American Chemical Society.

A common form of generating regulated 10–23 DNAzymes is with the use of multiplex or split designs, known as multicomponent DNAzymes (MNAzymes), which provide dual recognition and catalytic functions [74]. A complete MNAzyme consists of multiple “partzymes”, typically designated as partzyme A and partzyme B, each containing specific fragments of the catalytic core split at the T8 residue (Figure 5A). These partzymes assemble into a functional catalytic core only in the presence of specific target oligonucleotides that facilitate their hybridization. While many MNAzyme sensors have been developed based on the 10–23 core, the platform is highly modular, with some designs employing the split catalytic cores of different DNAzymes such as the 17E [75] or sodium‐dependant Na^+^‐BAS DNAzyme [76].

The concept of partial enzymatic fragments is not new. It dates back to 1958 with the discovery of ribonuclease‐S, a multi‐protein complex requiring two RNase‐A peptides for full enzymatic activity, with both fragments inactive when used independently [77]. The MNAzyme strategy is inspired by earlier split‐protein or enzyme systems like green fluorescent protein (GFP) and luciferase, which can be fused to ligand‐binding proteins and reconstitute their chromophore or catalytic activity upon target binding [78, 79]. These systems have been widely applied across numerous sensor platforms.

In contrast to split protein systems, MNAzymes operate through a dual‐recognition mechanism, giving them high modularity for the specific detection of viral RNA, miRNA, or other analytes. Although MNAzymes often exhibit lower catalytic activity compared to the intact 10–23 DNAzyme, their performance can be substantially enhanced through chemical modifications [56, 57, 80] or cationic polymers [81], which improve both stability and catalytic rates. Some MNAzyme‐based sensors have achieved attomolar LoDs when combined with target or signal amplification strategies as highlighted below. However, many designs require incubation at elevated temperatures (typically 50°C) to minimize unwanted background assembly of the MNAzyme—higher than the optimal reaction temperature of 37°C for unregulated 10–23 DNAzymes.

#### 3.2.1 Solution‐Based Colorimetric Sensors With 10–23 MNAzymes

Many solution‐based colorimetric sensors that utilize MNAzymes rely on the optical properties of AuNPs and are typically simple and inexpensive to implement. In one example, an MNAzyme‐based assay for *Salmonella choleraesuis* 16S rRNA detection employed AuNPs functionalized with a linker strand that could be cleaved by an MNAzyme in the presence of the target (Figure 5B) [82]. After isolating *S. choleraesuis* 16S rRNA using a total RNA extraction kit, the rRNA target facilitated the assembly of the two partzymes into a functional MNAzyme capable of cleaving the linker strand. When the linker strand remained intact, the AuNPs aggregated and produced a blue solution. By contrast, when the rRNA target was present, MNAzyme assembly and subsequent linker cleavage dispersed the AuNPs, yielding a red solution. Compared with the previously discussed colorimetric sensor for DENV detection using unregulated 10–23 DNAzymes, this assay demonstrated poorer overall performance, with lower sensitivity (LoD of 50 nM vs. 60 pM), higher incubation temperature (50°C vs. 37°C), and a longer assay time (30 min vs. 1.5 h) (Table 3). Similar MNAzyme optical sensors have shown improved performance for detecting sesame DNA in food samples [83] and synthetic DNA from various pathogens [84], achieving picomolar LoDs within 1.5 h at 50°C.

**TABLE 3:** Summary of split 10–23 DNAzyme (MNAzyme) biosensors.

10–23 DNAzyme type	Assay type	Target	Amplification step?	Clinical samples used?	Assay time	Assay temp.	LoD	Ref.
MNAzyme	Optical	*Salmonella* 16S rRNA	No	No	1.5 h	50°C	50 nM	[82]
MNAzyme	Optical	Sesame DNA	No	No	1.5 h	50°C	78 pM	[83]
MNAzyme	Optical	Synthetic DNA	No	No	1.5 h	50°C	50 pM	[84]
MNAzyme	Optical	MRSA DNA	RPA	Patient wound swab	3 h	37°C	33 aM	[85]
MNAzyme	LFD	Ochratoxin A	No	No	7 h	50°C	5 nM	[89]
MNAzyme (X10‐23 Pro)	LFD	SARS‐CoV‐2 mRNA	RPA	NPS	3 h	37°C	20 aM	[57]
MNAzyme	Fluorescence	*Chlamydia* DNA	No	No	1 h	50°C	10 pM	[93]
MNAzyme	Fluorescence	*Chlamydia* DNA	Cascade Amplification	No	30 min	50°C	1 fM	[94]
MNAzyme	Fluorescence	PD‐L1 RNA	No	No	1.5 h	50°C	50 fM	[95]
MNAzyme	Fluorescence	*Chlamydia* DNA	Hydrogel Cascade Amplification	No	25 min	50°C	50 fM	[96]
MNAzyme	Fluorescence	*tuberculosis* DNA	RPA	Sputum	1 h	50°C	3.3 aM	[97]
MNAzyme	Fluorescence	Anti‐digoxin antibodies	No	No	1.5 h	37°C	15 pM	[61]
MNAzyme	Fluorescence	Thrombin/Streptavidin	No	No	2.5 h	23°C	36 pM/1.3 pM	[100]
MNAzyme	Fluorescence (Microwell)	*Streptococcus and* adenovirus DNA	No	No	1.5 h	23°C	180 fM	[98]
MNAzyme (X10‐23 Pro)	Fluorescence (Digital droplet)	SARS‐CoV‐2 mRNA	No	NPS	3 h‐Overnight	34°C	3.7 fM	[99]
MNAzyme	Electrochemical	HPV mRNA	No	No	30 min	23°C	2.6 pM	[112]
MNAzyme	Electrochemical	Synthetic RNA	No	No	3 h	37°C	79 pM	[113]
MNAzyme	Electrochemical	MCF‐7 miRNA	RCA	No	5 h	37°C	1.66 fM	[114]

In another colorimetric MNAzyme sensor, functionalized AuNP linkers were combined with a target pre‐amplification step to improve the detection sensitivity of antibiotic resistance genes within Methicillin‐resistant *Staphylococcus aureus* (MRSA) DNA (Figure 5C) [85]. First, target DNA was extracted from MRSA in clinical samples using an automated DNA extraction instrument and subsequently amplified by RPA. The amplified target then facilitated assembly of the two partzymes into a complete MNAzyme, which cleaved the linker strand and dispersed the AuNPs. This assay achieved higher sensitivity compared to previous colorimetric solution‐based MNAzyme sensors and unregulated 10–23 DNAzyme systems, reaching an LoD of 33 aM. However, the need for an automated extraction platform and the elevated temperature (37°C) required for amplification increased assay complexity and resulted in a total assay time of approximately 3 h.

The use of isothermal pre‐amplification steps, such as RPA or RCA, is a common strategy for both regulated and unregulated 10–23 DNAzyme diagnostics to enhance sensitivity and specificity. Amplified products also offer strong compatibility with 10–23 DNAzymes, often serving as facilitators of MNAzyme formation or as trigger molecules for target‐activated 10–23 DNAzymes, described in Section 3.4. Preamplification can also help circumvent structural barriers in RNA targets, as described in Section 2.1.1. By reverse‐transcribing and amplifying a short fragment of a large, structured RNA target, the resulting amplicons can efficiently facilitate MNAzyme formation—as shown in the MRSA sensor—instead of being inefficiently cleaved by unregulated 10–23 DNAzymes.

Despite these advantages, preamplification increases assay cost and complexity, requiring additional reagents, enzymes, and incubation equipment. It also lengthens overall assay time, which may be undesirable in rapid POC settings. Thus, the decision to include a preamplification step must be carefully considered for each application.

#### 3.2.2 LFD‐Based Colorimetric Sensors With 10–23 MNAzymes

**FIGURE 6:**
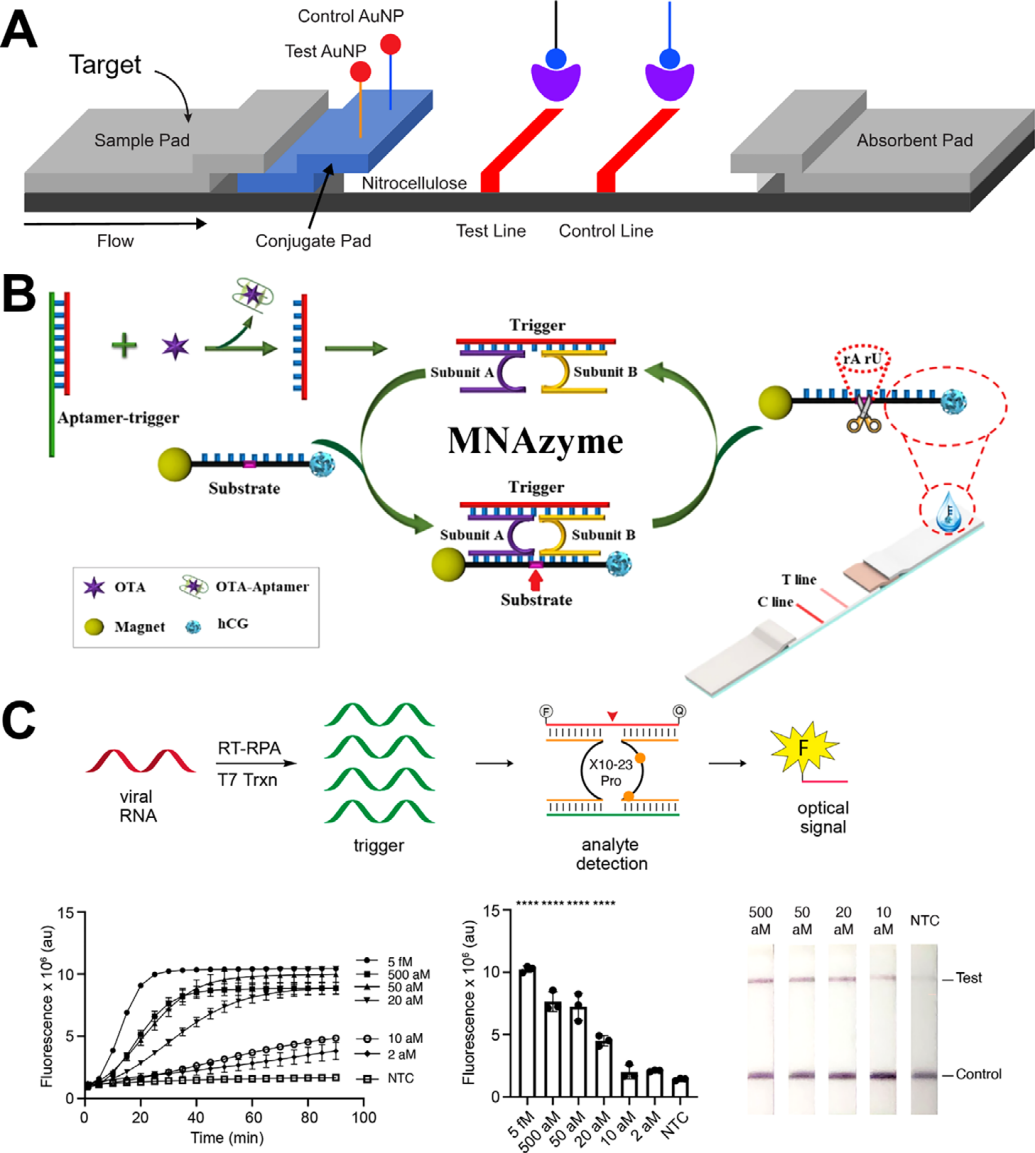
Lateral flow device (LFD)‐based sensors with MNAzymes. (A) Schematic of an LFD‐MNAzyme platform illustrating sample flow, AuNP conjugates, and test/control line readout. (B) LFD‐MNAzyme sensor for Ochratoxin A (OTA) detection, using a structure‐switching OTA aptamer as the trigger. Reproduced from Ref. [89] with permission. (C) LFD‐MNAzyme sensor for SARS‐CoV‐2 detection in which a reverse transcription recombinase polymerase amplification (RT‐RPA) step generates trigger sequences that activate a modified 10–23 DNAzyme to produce an optical readout. Reproduced with permission from Ref. [57]. Copyright 2025, American Chemical Society.

Lateral flow devices (LFDs) represent another widely used class of optical colorimetric sensors. They are highly modular and can be adapted for detecting a broad range of analytes, including insulin [86] and human chorionic gonadotropin (hCG) [87, 88]. A typical LFD comprises four main components: (1) a sample pad where the MNAzyme reaction mixture is applied; (2) a conjugate pad containing AuNP‐conjugated oligonucleotides for the test line (TL) and control line (CL); (3) a nitrocellulose membrane patterned with TL and CL capture probes; and (4) an absorbent pad (Figure 6A).

Because commercial pregnancy tests rely on antibody‐mediated capture of hCG and AuNP‐labelled antibodies to generate a visual readout, analogous MNAzyme/LFD assays can be constructed using hCG‐functionalized oligonucleotides, enabling rapid and user‐friendly detection of a variety of targets. In one example, a commercial pregnancy LFD was adapted to detect ochratoxin A (OTA) using a structure‐switching OTA aptamer as the molecular recognition element (Figure 6B) [89]. OTA was extracted from maize samples and incubated with the aptamer‐trigger complex, where OTA binding displaced the trigger strand. The released trigger promoted assembly of the complete MNAzyme, which cleaved an hCG‐functionalized RNA substrate immobilized on magnetic beads (MBs). After separation of the DNA–MB fragment, the liberated DNA–hCG fragment was introduced onto the LFD, producing a TL signal upon binding to immobilized anti‐hCG antibodies.

This sensor used MNAzyme‐mediated signal amplification, as each released trigger‐strand initiated cleavage of multiple substrate molecules through a feedback mechanism, yielding numerous DNA–hCG fragments. Despite this design, the reported LoD (5 nM) was relatively high compared to other MNAzyme‐based biosensors, and the overall assay required ∼5 h with multiple handling steps and incubations at 50°C. The limited sensitivity likely stems from the modest affinity of the OTA aptamer (*K*_D_ ≈360 nM) [90], which necessitates relatively high OTA concentrations for efficient recognition.

Although DNA‐based feedback amplification can enhance sensitivity, its effectiveness is influenced by trigger release efficiency and constrained by substrate hybridization kinetics, MNAzyme turnover, and product dissociation rates [22, 91]. Enzymatic pre‐amplification step (e.g., RPA or RCA) can further increase sensitivity, but adds to assay time, reagent cost, and operational complexity—trade‐offs that must be evaluated for each application.

Another MNAzyme‐based LFD sensor was developed for SARS‐CoV‐2 detection using an RT‐RPA pre‐amplification step to generate high concentrations of trigger strands, driving the assembly of an active MNAzyme (Figure 6C) [57]. The MNAzyme used in this assay—referred to as the RNA‐encoded viral nucleic acid analyte reporter (“REVEALR”)—was chemically modified with FANA (orange symbols) and threose nucleic acid (TNA) in both the catalytic core and substrate‐binding arms. This modified “X10‐23 Pro” MNAzyme displayed enhanced catalytic efficiency and improved resistance to exonuclease degradation in nasopharyngeal swab (NPS) samples compared with the unmodified form. Trigger binding enabled assembly of the active MNAzyme, which cleaved a biotinylated RNA reporter for LFD readout. Following heat inactivation of 12 positive and 11 negative patient NPS samples, RNA extraction (Viral RNA Mini Kit), RT‐RPA amplification, and X10‐23 Pro incubation, the LFD assay achieved a clinical accuracy of 100%, with an LoD of 20 aM and a total assay time of ∼ 3 h.

While LFDs provide rapid, simple, and low‐cost detection of pathogens, they face several limitations, including reliance on unstable reagents in pre‐amplification steps, limited quantitative capability, and the potential for cross‐reactivity in multiplex formats, which may constrain their broader applicability [92]. For applications requiring precise quantitation or highly multiplexed analysis, alternative diagnostic approaches may be more suitable.

Nonetheless, these examples highlight the simplicity of coupling MNAzyme‐based detection with LFD readouts for small‐molecule and nucleic acid targets. LFDs offer key advantages such as low cost, ease of use, and rapid signal generation compared with many other diagnostic platforms.

#### 3.2.3 Fluorescent Sensors With 10–23 MNAzymes

Many nucleic acid‐based biosensors employ fluorescence as the signal output because fluorescently labeled oligonucleotide probes can be readily integrated into MNAzyme designs and typically offer higher sensitivity than colorimetric assays.

**FIGURE 7:**
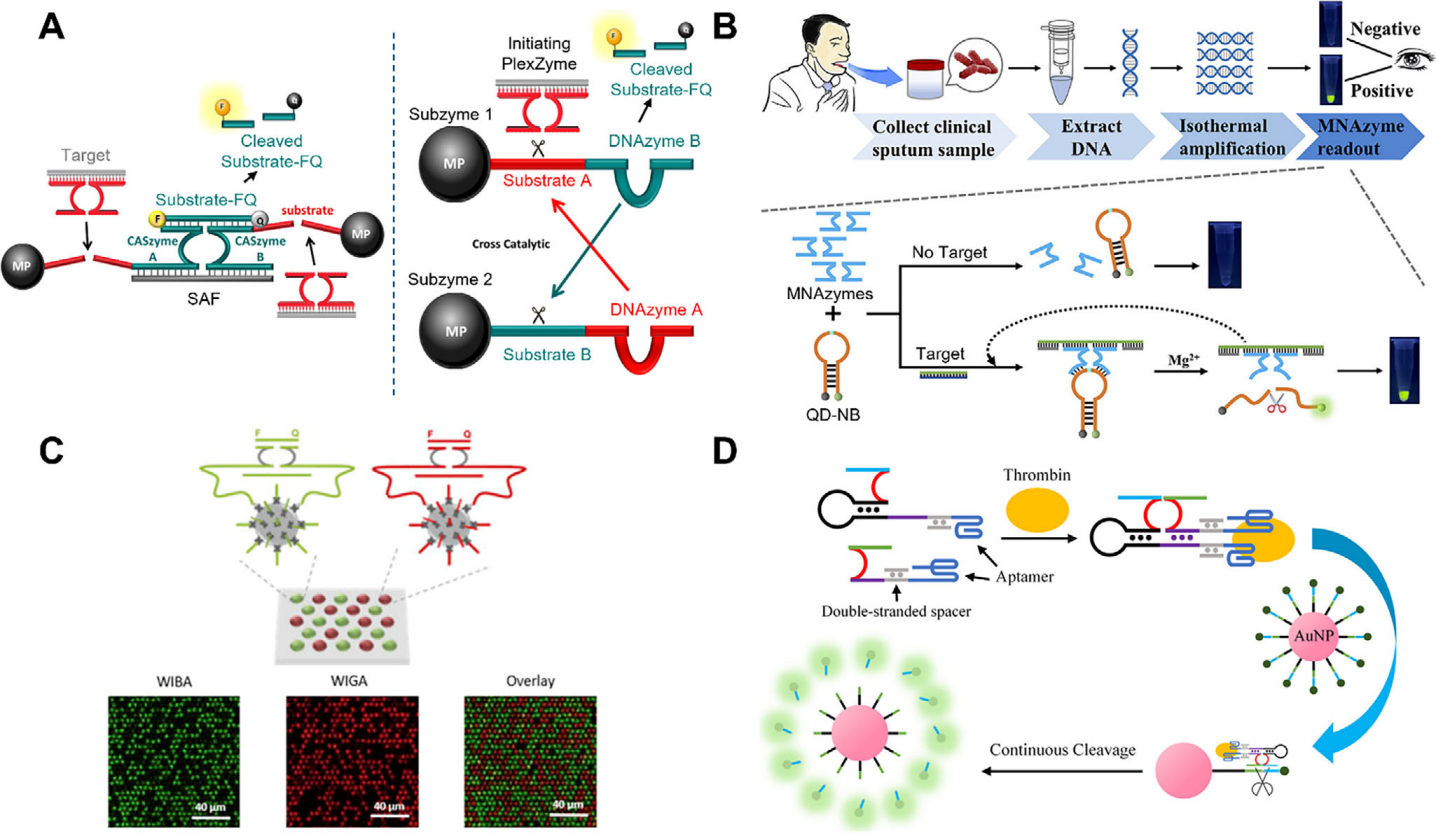
Fluorescence sensors with MNAzymes. (A) An MNAzyme‐based fluorescence sensor where the MNAzyme initiates cross‐reacting, magnetic microparticle (MP)‐functionalized “CASzymes” or subzymes in the presence of target, generating fluorescence. Reproduced from Refs. [93, 94] with permission. (B) MNAzyme detection of amplicons from preamplified clinical samples, where target binding assembles an active MNAzyme that cleaves an RNA‐containing quantum dot nano‐beacon (QD‐NB), producing fluorescence. Reproduced with permission from Ref. [97]. Copyright 2025 American Chemical Society. (C) Magnetic bead–functionalized partzymes designed to cleave separate fluorescence probes upon recognition of their respective targets, enabling multiplexed readout in a microwell format. Reproduced from Ref. [98] with permission. (D) Protein‐binding aptamer‐functionalized MNAzyme that assembles in the presence of target protein (e.g., thrombin), triggering continuous cleavage of a gold nanoparticle–conjugated fluorescent substrate. Reproduced from Ref. [100] with permission.

Two variations of a fluorescence sensor for *Chlamydia trachomatis* DNA detection have been reported, both using combinations of MNAzymes and 10–23 DNAzymes for target recognition and signal activation (Figure 7A, Left) [93]. In the first design, partzymes immobilized on magnetic microparticles (MPs)—referred to as “CASzymes”—were cleaved by an initiating MNAzyme in the presence of synthetic *C. trachomatis* DNA. Cleavage released the CASzymes from the MPs, and the supernatant was transferred to a separate chamber. There, the freed CASzymes hybridized to a signaling assembly facilitator (SAF), forming a complete MNAzyme that cleaved an FQ30 reporter substrate to generate fluorescence. Although the sensor achieved an LoD of 10 pM within an hour at 50°C, it required multiple separation and transfer steps, limiting its compatibility with simple POC settings.

To further improve sensitivity, a second design used a cascade of cross‐reacting “Subzymes”—single‐stranded oligonucleotides containing a complete 10–23 catalytic core and a complementary RNA substrate (Figure 7A, Right) [94]. In this system, an MNAzyme cleaved Subzyme 1 in the presence of target, releasing DNAzyme B, which cleaved an FQ30 reporter to generate fluorescence. DNAzyme B also cleaved Subzyme 2, releasing DNAzyme A, which then cleaved Subzyme 1 to produce more DNAzyme B, creating a cross‐reactive amplification loop. Both subzymes were immobilized on MPs to suppress background amplification, and reaction chambers were separated by porous membrane bags (PMBs). Freed DNAzyme B diffused through the PMBs and initiated cleavage of Subzyme 2. This system improved the LoD by ∼10 000‐fold compared to the original CASzyme design, enabling detection within 30 min at 50°C. A related subzyme platform was later applied to detect programmed cell death ligand 1 (PD‐L1) RNA from cancer cell lines, reaching an LoD of 50 fM in 1.5 h at 50°C [95].

Harnessing the intrinsic cross‐reactivity of 10–23 DNAzymes provides a straightforward amplification strategy that avoids polymerase enzymes. However, PMB‐based workflows are difficult to implement in POC contexts because they require multiple incubation, separation, and pipetting steps. To simplify the format, other groups developed subzyme cascades embedded in solid hydrogel matrices. In one example, subzymes were crosslinked to acrydite‐modified strands to form a polyethylene glycol diacrylate network [96]. MNAzyme activation in the presence of target destabilized the hydrogel, releasing additional subzymes and increasing fluorescence. This sensor achieved an LoD of 50 fM with a total assay time of 25 min at 50°C without centrifugation or magnetic bead (MB) handling.

As with other MNAzyme sensors, isothermal pre‐amplification can be used to enhance fluorescence‐based detection. In an assay designed for *Mycobacterium tuberculosis* (MTB) detection, sputum samples were processed using an automated nucleic acid extractor, followed by RPA amplification for 20 min (Figure 7B) [97]. The amplicons were then incubated with MNAzymes and an RNA‐containing quantum dot DNA hairpin nano‐beacon probe (QD‐NB). Cleavage of the QD‐NB by the active MNAzyme produced a fluorescent signal that could be visualized under ultraviolet light. The assay detected MTB genomic DNA in clinical samples with 100% accuracy (36 positive and 20 negative samples), achieving an LoD of 3.3 aM within 1 h at 50°C.

Fluorescence readouts are also readily adapted to multiplex formats. One example used MB–coupled MNAzymes to detect genomic DNA from *Streptococcus pneumoniae* and human adenovirus from spiked NPS samples using a microwell fluorescence platform (Figure 7C) [98]. Two distinct MNAzymes were designed to recognize each target and cleave separate fluorogenic probes labeled with HEX or FAM. After sample addition, fluorescence was quantified using an inverted fluorescence microscope with appropriate filter sets. The assay achieved an LoD of 180 fM at room temperature within 1.5 h, without requiring pre‐amplification, although the need for specialized microscopy equipment restricts its use to laboratory settings.

A similar multiplexing strategy was demonstrated using the chemically modified X10‐23 Pro MNAzyme in the REVEALR system. Microfluidic droplet generation increased the effective concentration of target molecules, enabling highly sensitive detection [99]. Genomic RNA from COVID‐19 patient NPS samples was extracted and analyzed without preamplification, yielding an LoD of 3.7 fM and a clinical sensitivity of 95%, but required overnight incubation, which is not ideal for rapid diagnostics.

MNAzymes can also be adapted for protein detection by functionalizing partzymes with small‐molecule antigens. In one example, digoxin‐conjugated partzymes enabled detection of anti‐digoxin antibodies in spiked serum samples [61]. After antibody recognition, partzyme assembly produced a complete MNAzyme that cleaved circular crRNA, activating Cas12a and generating a fluorescent signal with an LoD of 15 pM at 37°C within 1.5 h.

In another design, partzymes were conjugated to subunits of protein‐binding aptamers (Figure 7D) [100]. Target binding facilitated hybridization of the aptamer subunits, assembling a catalytically active MNAzyme capable of detecting streptavidin and thrombin with LoDs of 1.3 and 36 pM, respectively, after 2.5 h at room temperature. However, this strategy is limited by the availability of aptamers with high affinity and the requirement for two aptamers to bind the same target (i.e., a sandwich configuration), restricting its use to proteins with multiple accessible epitopes such as thrombin [101, 102].

A notable feature of this assay is that the MNAzyme also functioned as the SRE, acting as a molecular motor that cleaved an AuNP‐functionalized, fluorescently labeled substrate upon target binding [103, 104]. Using AuNPs improves signal generation through fluorescence quenching, eliminating the need for a dedicated quencher (as required in the FQ30 substrate) [105]. Additionally, AuNP‐conjugated oligonucleotides exhibit reduced susceptibility to nuclease degradation, improving sensor robustness in biological samples [106].

In summary, numerous fluorescent sensors have been developed using MNAzymes to detect a wide range of analytes. Fluorescence provides high analytical sensitivity—often without requiring pre‐amplification—due to its superior signal‐to‐noise characteristics compared with colorimetry. However, fluorescence‐based assays are typically less suited for rapid POC applications due to their need for specialized equipment and multi‐step processing. Recent advances in portable fluorescence readers, such as the commercial “ANDalyze” platform (Alpha Measurement Solutions) for heavy‐metal detection and emerging devices for nucleic acid [107] and small molecule [108] sensing, may help bridge this gap and enable wider adoption of fluorometric MNAzyme‐based diagnostics.

#### 3.2.4 Electrochemical Sensors With 10–23 MNAzymes

Electrochemical biosensors quantify target recognition by generating an electrical signal proportional to analyte concentration. MNAzymes have been widely incorporated into electrochemical platforms because they support quantitative measurements, high sensitivity with small sample volumes, and minimal sample preparation across diverse molecular targets [109, 110, 111].

**FIGURE 8:**
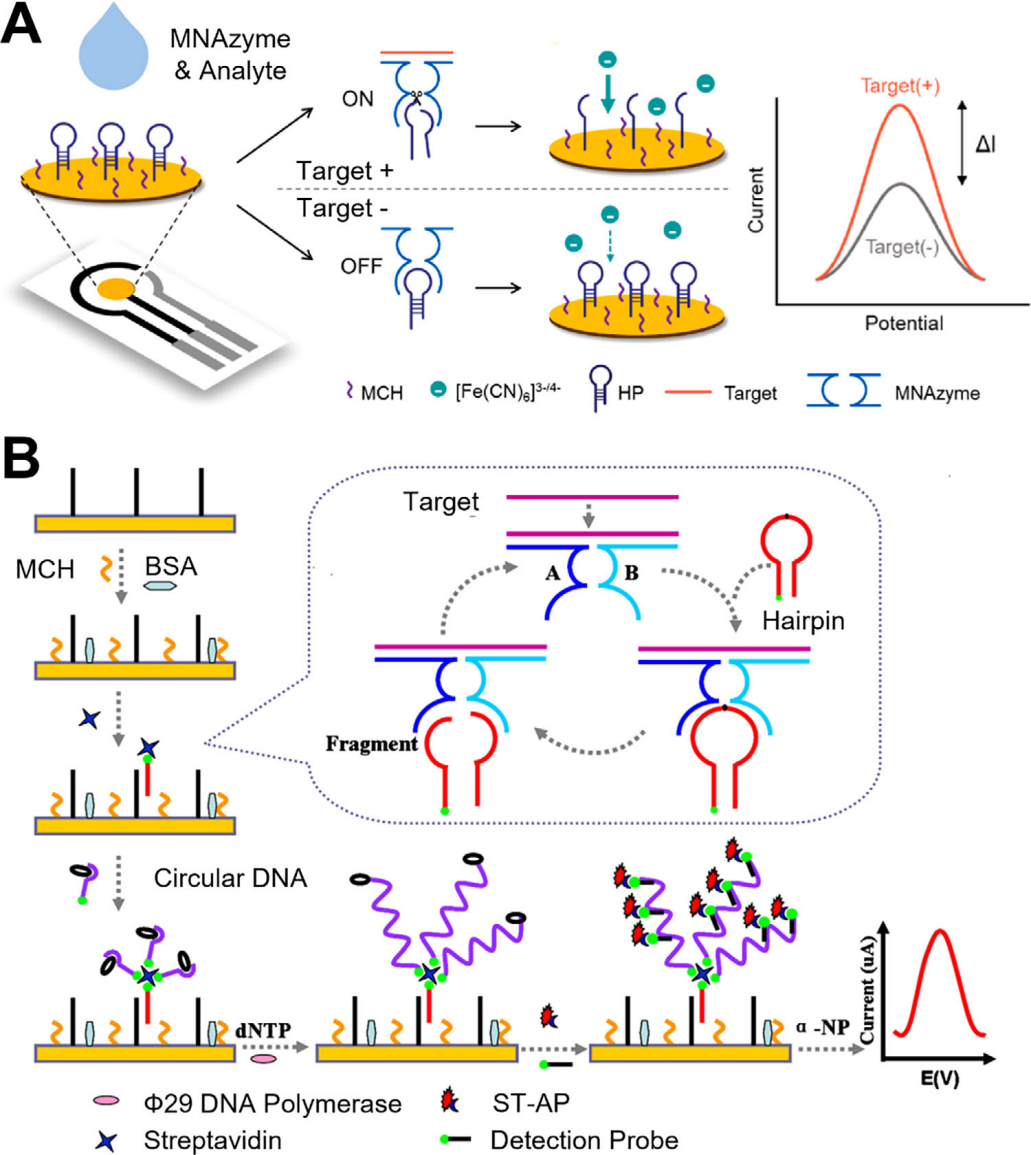
Electrochemical sensors with MNAzymes. (A) Individual partzymes assemble into an active MNAzyme in the presence of target, cleaving an embedded RNA linkage within a DNA hairpin probe. This cleavage increases the mobility of the ferricyanide redox indicator at the electrode surface, producing an elevated electrochemical current. Reproduced from Ref. [112] with permission. (B) In a related design, an active MNAzyme cleaves a biotinylated hairpin probe that can be captured on the electrode surface, where it binds to streptavidin and initiates rolling circle amplification (RCA), enhancing the electrochemical signal. Reproduced from Ref. [114] with permission.

An early example employed an MNAzyme–hairpin substrate immobilized on a gold electrode for detection of HPV mRNA in spiked total RNA extracted from HeLa cells (Figure 8A) [112]. Presence of the target mRNA induced assembly of the active MNAzyme, which cleaved the hairpin substrate and reduced the impedance of the ferricyanide redox indicator at the electrode surface, thereby increasing electrochemical current. The assay achieved an LoD of 2.6 pM within 30 min at room temperature and was compatible with rapid measurements using a portable USB‐powered electrochemical detector.

Two subsequent miRNA sensors used MNAzymes to cleave a biotinylated hairpin substrate, enabling hybridization of the cleaved fragment to capture probes on a gold electrode [113]. Using this direct, amplification‐free format, synthetic RNA was detected with an LoD of 79 pM in 3 h at 37°C. In the second sensor, an RCA amplification step was incorporated after MNAzyme‐mediated target recognition to improve sensitivity (Figure 8B) [114]. First, streptavidin was added, linking the biotinylated hairpin to a second biotinylated circular DNA probe. Next, Phi29 DNA polymerase generated long DNA concatemers that acted as templates for the detection probe, and a streptavidin–alkaline phosphatase (ST–ALP) reporter enabled electrochemical detection via dephosphorylation of the α‐naphthyl phosphate (α‐NP) redox substrate. This approach detected purified miRNA‐21 from human breast adenocarcinoma MCF‐7 cells with an improved LoD of 1.66 fM at 37°C, though the assay required a 5‐h incubation due to the non‐exponential nature of linear RCA.

The adoption of electrochemical POC sensors is expected to increase as instrumentation continues to miniaturize. Whereas earlier assays relied on expensive benchtop potentiostats, recent advances include compact power sources and portable potentiostats—such as the USB adaptor used in the HPV MNAzyme sensor and other electrochemical DNAzyme assays [115, 116]—which improve portability, reduce cost, and simplify operation. Nevertheless, most electrochemical 10–23 DNAzyme sensors still require multiple washing steps to remove components that can contribute to background signal, complicating workflows. Additionally, many reported assays rely on multi‐step and labor‐intensive electrode functionalization, which can lead to batch‐to‐batch variability. Electrodes are also susceptible to surface fouling, limiting their reusability; while disposable electrodes circumvent this issue, they increase overall assay cost.

Taken together, electrochemical sensors incorporating 10–23 DNAzymes have strong potential for rapid, sensitive, and quantitative molecular detection, but further development is needed to improve their simplicity, robustness, and cost‐effectiveness for practical diagnostic use.

### 3.3 Target‐Activated 10–23 DNAzyme Sensors

**FIGURE 9:**
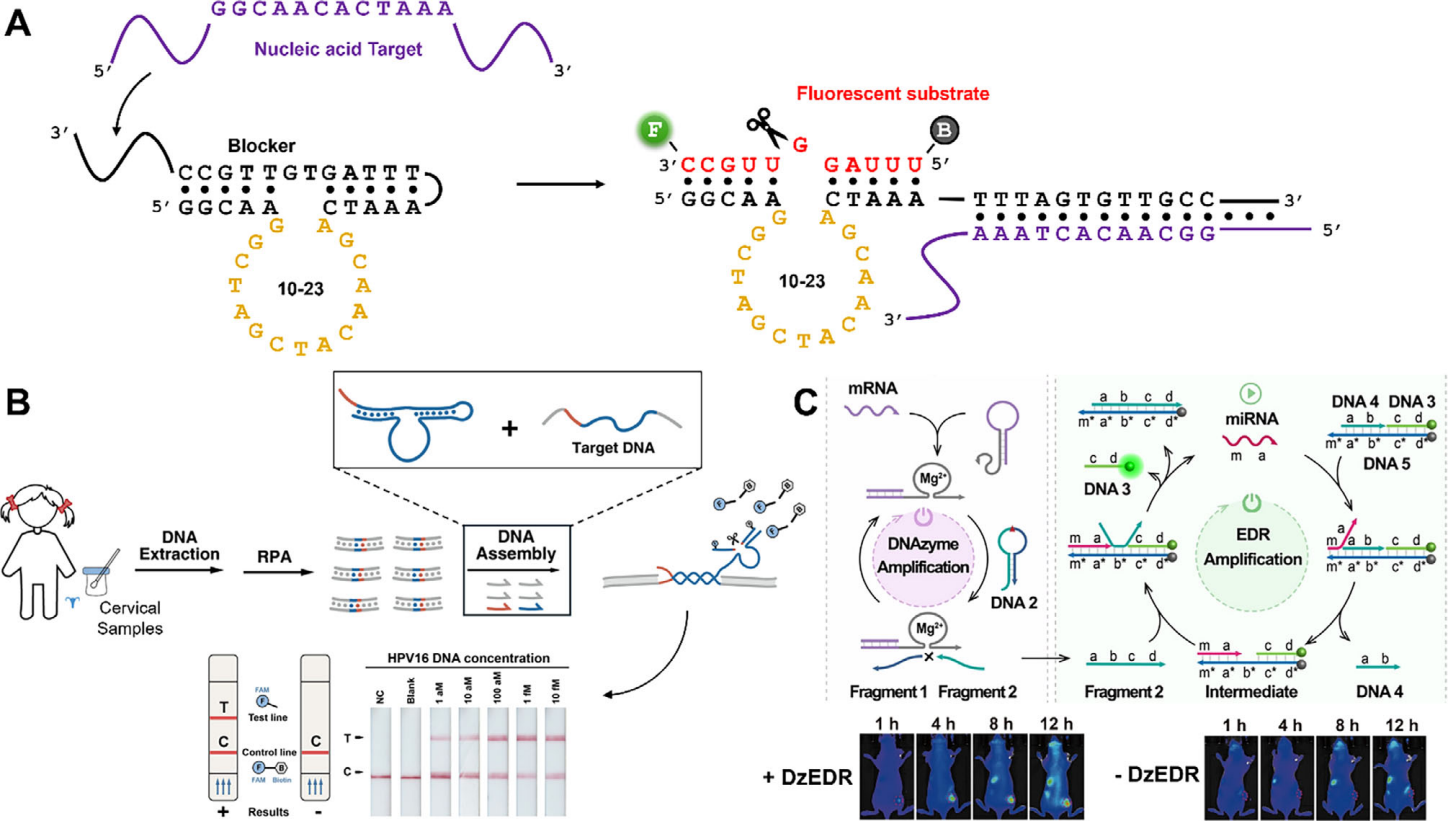
Target‐activated 10–23 DNAzyme sensors. (A) A caged, *cis*‐acting 10–23 DNAzyme in which a blocker domain prevents substrate recognition. Hybridization of a target oligonucleotide to the toehold region initiates strand displacement, releasing the blocker and activating the DNAzyme. (B) Schematic of CLARISSA, a target‐activated 10–23 DNAzyme assay for HPV DNA. After RPA amplification, HPV DNA activates the caged DNAzyme via TMSD, enabling cleavage of a biotinylated RNA substrate that is detected by lateral flow analysis. Reproduced from Ref. [119] with permission. (C) Design of an intracellular sensor for miRNA‐21 using an entropy‐driven reaction (EDR) amplification system. Cell‐specific mRNA first activates the caged 10–23 DNAzyme, producing a cleaved fragment that fuels cyclic TMSD to release a fluorescent probe. Reproduced from Ref. [120] with permission.

Target‐activated sensors rely on a DNA oligonucleotide that shares similar sequence elements to the substrate‐recognition arms of the 10–23 DNAzyme and functions as a “blocker” to prevent the DNAzyme from hybridizing to—and thus cleaving—its intended RNA substrate. These blocked or “caged” 10–23 DNAzymes can operate in a *cis* configuration (Figure 9A), where the blocking sequence is part of the same strand as the DNAzyme, or in *trans*, where the blocker and DNAzyme exist as two separate oligonucleotides. Activation occurs through a toehold‐mediated strand displacement (TMSD) reaction, in which the target nucleic acid hybridizes to a single‐stranded toehold region on the blocker, displacing it from the DNAzyme and freeing the binding site for substrate recognition and cleavage.

Some of the earliest and simplest target‐activated sensors were solution‐based designs that used TMSD to liberate G‐quadruplex–forming DNAzymes (similar to the unregulated 10–23‐coupled SDA sensor described in Section 2.1). These G‐quadruplex DNAzymes generated a visible colorimetric signal in the presence of hemin and peroxide [117, 118]. Although these assays produce easily recognizable readouts, their detection sensitivity is relatively modest—typically in the nanomolar to picomolar range—and they often require long incubation times and reagents with limited shelf stability, reducing their practicality for diagnostic use. Since then, several other sensors have been developed using target‐activated 10–23 DNAzymes in combination with LFDs, electrochemiluminescence and intracellular sensing methods.

#### 3.3.1 Target‐Activated 10–23 DNAzymes as Lateral Flow Sensors

Several groups have developed target‐activated 10–23 DNAzyme sensors that combine isothermal amplification with alternative signal‐output strategies to improve detection sensitivity. A recent example paired RPA with a blocked 10–23 DNAzyme for HPV DNA detection using an LFD readout, termed the CRISPR‐like assay using RNA‐cleaving DNAzyme coupled with isothermal sequence and signal amplification (CLARISSA) (Figure 9B) [119]. Cervical swab samples were processed to extract HPV DNA, which was then amplified using RPA. The amplified HPV DNA activated the caged 10–23 DNAzyme via TMSD, enabling cleavage of a biotinylated RNA substrate. The cleaved substrate was subsequently applied to an LFD and captured on the test line. The assay achieved an LoD of 10 aM and demonstrated 100% clinical sensitivity and 97.4% specificity across 189 clinical samples (Table 4).

**TABLE 4:** Summary of target‐activated 10–23 DNAzyme biosensors.

10–23 DNAzyme type	Assay type	Target	Amplification step?	Clinical samples used?	Assay time	Assay temperature	LoD	Ref.
Target‐activated	LFD	HPV DNA	RPA	Cervical Swab	1 h	37°C	10 aM	[119]
Target‐activated	LFD	miRNA‐155	No	Patient Serum	5 h	28°C	32 fM	[121]
Target‐activated	Fluorescence (in vivo)	miRNA‐21	EDR	No (in vivo)	12 h	Physiological	3 pM	[120]
Target‐activated	Fluorescence (in vivo)	miRNA‐21	RCA	No (in vivo)	4 h	Physiological	7.2 fM	[127]
Target‐activated	ECL	HIV DNA	No	No	2 h	37°C	0.82 fM	[125]
Target‐activated	ECL	miRNA‐141	No	No	30 min	37°C	53 fM	[126]

A similar target‐activated 10–23 DNAzyme LFD assay was developed for detecting serum‐derived miRNAs but did not incorporate a pre‐amplification step [121]. This assay, termed the sensitive loop‐initiated DNAzyme biosensor for nucleic acid detection (SPOT), detected miRNA‐155 extracted from serum with an LoD of 32 fM and achieved 100% clinical sensitivity and specificity across 16 patient samples. However, visible test‐line bands were also observed in negative control LFDs, complicating rapid interpretation of true positives without optical‐density measurement and statistical analysis. Similar issues were reported for additional targets, including miRNA‐21 and SARS‐CoV‐2 RNA, where false‐positive test‐line signals were likewise observed in negative controls.

#### 3.3.2 Target‐Activated 10–23 DNAzymes as Electrochemiluminescence Sensors

Target‐activated 10–23 DNAzymes have also been incorporated into electrochemiluminescence (ECL) sensing platforms. ECL sensors offer inherently high sensitivity because they operate without an external light source, minimizing background from light scattering and improving signal‐to‐noise ratios [122, 123, 124].

In one example, a synthetic HIV DNA target hybridized to a blocked 10–23 DNAzyme, but instead of activating the DNAzyme through TMSD, target hybridization recruited Exonuclease III. Exonuclease III degraded the blocker strand, thereby freeing the DNAzyme for catalytic activity. The activated DNAzyme cleaved an ECL‐emitter‐labeled substrate hybridized to the electrode surface; its removal reduced the ECL signal in this “turn‐off” design. The assay achieved an LoD of 0.82 fM after 2 h of incubation at 37°C [125]. In a similar ECL sensor for miRNA‐141, both the 10–23 and 8–17 DNAzymes were used, yielding an LoD of 53 fM at 37°C within only 30 min [126].

With only two reports to date, ECL platforms using 10–23 DNAzymes remain at the proof‐of‐concept stage, and no portable devices or clinical samples have yet been evaluated. As with electrochemical 10–23 sensors, these assays require multiple washing steps and specialized equipment, and they depend on tight control of the electrochemical environment to maintain DNAzyme activity. Nevertheless, integrating DNAzymes with ECL readouts remains a promising direction for developing highly sensitive nucleic‐acid sensors due to the inherently low background of ECL detection.

#### 3.3.3 Target‐Activated 10–23 DNAzymes as Fluorescent Intracellular Sensors

An emerging application of target‐activated 10–23 DNAzymes is their use in intracellular fluorometric sensing. In this approach, intracellular nucleic acid targets (DNA or RNA) activate a caged 10–23 DNAzyme, which then cleaves a fluorogenic substrate, generating a measurable increase in fluorescence.

One example monitored miRNA‐21 in mice by coupling target‐activated 10–23 DNAzymes with an entropy‐driven reaction (EDR) amplification system, essentially a cyclic TMSD process (Figure 9C) [120]. The sensor used a dual‐recognition mechanism: first, cell‐specific mRNA activated the caged DNAzyme, cleaving an embedded RNA linkage within a hairpin probe. In a separate step, the target miRNA hybridized to a duplex fluorescence reporter probe via TMSD, exposing a binding site for the cleaved fragment. The fragment then served as fuel for EDR amplification, displacing a fluorescent probe from the duplex via TMSD. In vivo, the assay detected miRNA‐21 at concentrations as low as 3 pM in tumors of transfected mice after 12 h. Another intracellular miRNA‐21 sensor used RCA amplification instead of EDR, improving the LoD to 7.2 fM after 4 h [127], highlighting the advantages of enzymatic amplification over non‐enzymatic methods for more sensitive detection.

Several other 10–23 DNAzyme sensors have also been developed for intracellular Mg^2+^ sensing in cancer cells, given the high activity and selectivity with Mg^2+^. In one example, a caged 10–23 DNAzyme was activated following cleavage by a restriction endonuclease present in HeLa cells, I‐SceI. The activated 10–23 DNAzyme cleaved a fluorescent probe, producing a signal with an LoD of 2.1 mM after a 24–48 h incubation period [128]. In another intracellular Mg^2+^ sensor, a caged 10–23 DNAzyme was inactivated using a DNA modulator containing an abasic site. Cleavage of the abasic site by APEI – present in cancer cells—activated the 10–23 DNAzyme, facilitating cleavage of a fluorescent probe. While the initial design used an Zn^2+^‐dependent DNAzyme with an LoD of 600 nM after 4 h post injection, the detection limit for Mg^2+^ using the 10–23 DNAzyme was not reported [129].

While target‐activated 10–23 DNAzymes are common as intracellular sensors, other designs exist, including co‐transfection of the 10–23 DNAzyme with GFP and RFP‐encoded plasmids for Mg^2+^ detection in HeLa cells. In the presence of Mg^2+^, GFP mRNA was cleaved by the 10–23 DNAzyme, generating only RFP [130]. Overall, the scope is quite limited, as the 10‑23 DNAzyme exhibits strong selectivity only for Mg^2^^+^ and performs poorly with other divalent cations [131]. In addition, an effective sensor should have an LoD for Mg^2^^+^ below the physiological concentration of 0.5 mM [132, 133], whereas the sensors described here exist at, or above, that threshold.

Moving forward, broader adoption of 10–23 as an intracellular sensor will require addressing challenges such as improving activity under low Mg^2+^ concentrations and reducing susceptibility to endonuclease degradation. Progress has been made using chemically modified 10–23 DNAzymes [51, 54, 56] and AuNP‐conjugated DNAzymes [106, 134], both of which enhance stability and catalytic performance in cellular environments. The reader is encouraged to refer to these reviews for a more in‐depth analysis of intracellular sensing with DNAzymes [135, 136].

### 3.4 Incorporation of 10–23 Into Amplification Products

Embedding an active 10–23 DNAzyme sequence into primers or amplification products enables PCR or isothermal amplification reactions to generate DNAzymes in situ, which can then cleave fluorescent or colorimetric probes upon formation. Examples include incorporating the 10–23 DNAzyme into qPCR assays for Kras DNA detection [137] and into RT‐qPCR for PML/RARα mRNA transcripts [138]. These strategies provide highly sensitive and direct readouts of amplified products, improving specificity and potentially reducing the need for separate probe‐based chemistries. However, despite their analytical promise, no POC sensing platforms have yet been developed using these approaches.

## 4 Challenges and Outlook

Despite the remarkable specificity, catalytic efficiency, and programmability of the 10–23 DNAzyme, many of the biosensing and diagnostic strategies described in this review still face significant obstacles that limit their broad adoption and translation into practical applications. This section outlines the major limitations of 10–23 DNAzyme–based sensing systems and discusses how emerging approaches may help overcome these barriers.

### 4.1 Performance of 10–23 in Biosensing and Diagnostics

Under optimal conditions—appropriate pH, temperature, Mg^2^^+^ concentration, and accessible RNA substrate structure—the 10–23 DNAzyme exhibits some of the highest catalytic rates among RNA‐cleaving DNAzymes. However, deviations from these conditions frequently impair cleavage efficiency, resulting in reduced sensitivity and slower assay kinetics in practical biosensing scenarios. Several mitigation strategies described in this review aim to address these limitations.

Chemical modification of the substrate‐recognition arms or catalytic core with XNAs can enhance both catalytic activity and resistance to nuclease degradation, thereby stabilizing the DNAzyme in complex biological samples. The FANA‐based “X10‐23 Pro” MNAzyme in the REVEALR system [57] is one prominent example, and other groups have explored 2′‐Me [139], LNA [55, 140], 2′‐OMe [141] and TNA [142] for similar purposes. Although effective, these modifications increase synthesis complexity and cost, limiting widespread implementation.

Immobilization of the 10–23 DNAzyme or SRE on AuNPs can improve stability and reduce degradation in biological matrices, as demonstrated in the colorimetric DENV sensor [41]. However, this approach does not eliminate degradation entirely and may not generalize across all assay formats [106]. Temperature remains another significant constraint: while 10–23 and MNAzymes function optimally near 37 °C [13] and 50°C [74], respectively, many point‐of‐care biosensors must operate at or below room temperature (23°C) [143, 144] to avoid heating equipment. Adjusting recognition arm length and other parameters can improve low‐temperature activity, but catalytic performance still drops substantially in most cases.

Similarly, the 10–23 DNAzyme's dependence on relatively high Mg^2^^+^ concentrations (15–25 mM) [132, 145] limits its use in biological samples or intracellular environments, where free Mg^2^^+^ levels are typically <2 mM. XNA‐modified variants show improved activity under low‐Mg^2^^+^ conditions [51], but the trade‐off between synthesis cost and operational benefit must be considered.

Finally, effective cleavage of large, structured RNA targets—such as viral genomes—remains challenging [43]. Strategies such as Mg^2^^+^ supplementation [146], XNA incorporation [47, 48, 49], or ASO assistance [44, 46] can improve target accessibility, but each introduces practical limitations for assay simplicity, cost, or robustness.

### 4.2 Sample Quality and Clinical Validation

Real‐world biological samples contain inhibitors, nucleases, and structurally complex nucleic acids that can markedly reduce cleavage efficiency. While many published sensors rely on synthetic or spiked samples to demonstrate analytical performance, these systems often perform differently when challenged with patient samples, which exhibit substantial variability in viscosity, matrix composition, and target abundance [147].

Among the 31 biosensing platforms reviewed here (summarized in Tables 1, 2, 3, 4), only eight have been validated with clinical specimens—including NPS, saliva, cervical swabs, and wound swabs—highlighting the substantial gap between proof‐of‐concept demonstrations and real diagnostic deployment.

### 4.3 Sample Processing Constraints

Although rigorous sample purification improves nucleic‐acid quality and reduces background signals, it increases assay cost and complexity, often requiring commercial kits, automated extractors, or centrifugation steps incompatible with POC workflows. Many 10–23 biosensors that perform well with synthetic targets will therefore require redesign and optimization when applied to minimally processed clinical samples.

### 4.4 Practical Challenges for Achieving ASSURED Standards

Even with improvements to catalytic performance, many 10–23 DNAzyme biosensing formats still struggle to meet the WHO ASSURED criteria. Temperature control is a dominant limitation: 25 of the 31 reported biosensors require incubation above room temperature. Additionally, although preamplification steps (e.g., RPA, RCA) powerfully enhance analytical sensitivity, they often extend assay time and require added enzymes, undermining simplicity and rapid turnaround.

Overall, while the 10–23 DNAzyme has demonstrated impressive versatility a‐cross biosensing platforms, substantial engineering and validation work remains necessary to transition these systems into deployable diagnostic tools.

## 5 The Future of 10–23 DNAzymes in Biosensing and Diagnostics

A promising direction for the field is the development of RNA‐cleaving DNAzymes that exceed the catalytic performance of 10–23. Although 10–23 remains the fastest naturally derived RCD, its maximal rate constant (∼12 min^−^^1^) remains orders of magnitude lower than that of protein enzymes such as RNase A (∼10^5^ min^−^^1^) [148]. Future in vitro selection efforts could incorporate shorter reaction windows, lower substrate concentrations, and more stringent selection pressures to enrich for DNAzymes with higher catalytic efficiency and improved substrate accessibility. Once top candidates emerge, subsequent selection rounds can refine their performance under realistic biosensing conditions—low Mg^2+^ environments, complex biological matrices, and room‐temperature operation.

Recent studies have demonstrated the viability of evolving DNAzymes directly in physiological fluids, such as undiluted human serum, underscoring the potential to select catalysts inherently compatible with challenging sample types [149]. Pre‐structured libraries that incorporate motifs from high‐affinity aptamers or highly active DNAzymes have also yielded improved catalysts [150], including XNA‐based DNAzymes with enhanced stability and nuclease resistance [54, 151]. These innovations collectively point toward a future in which next‐generation RCDs significantly outperform 10–23 in both speed and robustness.

While the 10–23 DNAzyme has already enabled a diverse array of biosensing and diagnostic formats, critical challenges remain before its full potential can be realized. Enhancing catalyst stability, expanding temperature tolerance, reducing Mg^2+^ dependence, and validating sensors with real clinical samples are essential steps toward platforms that satisfy WHO ASSURED criteria. With ongoing advances in nucleic acid chemistry and integrated assay design, the 10–23 DNAzyme—and its emerging successors—are well positioned to play an increasingly influential role in next‐generation biosensing and diagnostic technologies.

## Conflicts of Interest

The authors declare no conflicts of interest.

## Data Availability

The data that support the findings of this study are available from the corresponding author upon reasonable request.
